# Practice Patterns of Screening for Hydroxychloroquine Retinopathy in South Korea

**DOI:** 10.1001/jamanetworkopen.2023.14816

**Published:** 2023-05-23

**Authors:** Jiyeong Kim, Ko Eun Kim, Ji Hong Kim, Seong Joon Ahn

**Affiliations:** 1 Biostatistical Consulting and Research Lab, Industry-University Cooperation Foundation, Hanyang University, Seoul, Republic of Korea; 2 Department of Ophthalmology, Asan Medical Center, Ulsan University College of Medicine, Seoul, Republic of Korea; 3 Department of Ophthalmology, Hanyang University Hospital, Hanyang University College of Medicine, Seoul, Republic of Korea

## Abstract

**Importance:**

Practice patterns of hydroxychloroquine retinopathy screening have not yet been reported at a national level in South Korea.

**Objective:**

To investigate the practice patterns of timing and modality for hydroxychloroquine retinopathy screening in South Korea.

**Design, Setting, and Participants:**

This nationwide population-based cohort study of patients in South Korea used data from the national Health Insurance Review and Assessment database. Patients at risk were identified as those who had initiated hydroxychloroquine therapy between January 1, 2009, and December 31, 2020, and used it for 6 months or more. Patients were excluded if they underwent any of the 4 screening modalities recommended by the American Academy of Ophthalmology (AAO) for other ophthalmic diseases prior to hydroxychloroquine use. The timing and modalities of screening used in baseline and monitoring examinations were assessed between January 1, 2015, and December 31, 2021, among patients at risk and long-term (≥5 years) users.

**Exposure:**

Adherence of baseline screening practices to the 2016 AAO recommendations (fundus examination within 1 year of drug use) was evaluated; adherence of monitoring examinations in year 5 was classified as appropriate (≥2 tests recommended by the AAO), unscreened (no test performed), and underscreened (insufficient number of tests).

**Main Outcomes and Measures:**

Timing of screening and modalities used at baseline and monitoring examinations.

**Results:**

A total of 65 406 patients at risk (mean [SD] age, 53.0 [15.5] years; 50 622 women [77.4%]) were included; 29 776 patients were long-term users (mean [SD] age, 50.1 [14.7] years; 24 898 women [83.6%]). Baseline screening was performed for 20.8% of the patients within 1 year, with a gradual increase from 16.6% in 2015 to 25.6% in 2021. Monitoring examinations, mostly using optical coherence tomography and/or visual field tests, were performed for only 13.5% of the long-term users in year 5 and for 31.6% of the long-term users after 5 years. Appropriate monitoring was performed for less than 10% of long-term users each year from 2015 to 2021; however, the percentage gradually increased over time. The percentage of patients undergoing any monitoring examination in year 5 was 2.3 times greater for those who had received baseline screening than for those who did not (27.4% vs 11.9%; *P* < .001).

**Conclusions and Relevance:**

This study suggests there is an improving trend in retinopathy screening among hydroxychloroquine users in South Korea; however, most long-term users remained unscreened after 5 years of use. Baseline screening may be useful in reducing the number of unscreened long-term users.

## Key Points

**Question:**

What are the screening practices for hydroxychloroquine retinopathy in South Korea?

**Findings:**

This nationwide population-based cohort study of 65 406 patients at risk of retinopathy (29 776 long-term users of hydroxychloroquine) using national health insurance claims data found that the annual number of hydroxychloroquine users increased from 2009 to 2020; baseline screening was performed for 20.8% of the patients within 1 year, with a gradual increase from 16.6% in 2015 to 25.6% in 2021. Annual percentages of patients receiving appropriate screening for retinopathy showed an improving trend for both baseline and monitoring examinations; those who underwent baseline screening received monitoring examinations more frequently than those who did not.

**Meaning:**

This study suggests that most long-term hydroxychloroquine users in South Korea have not undergone screening for retinopathy after 5 years of use; however, baseline screening may be useful in reducing the number of unscreened long-term users.

## Teaser

This cohort study uses national health insurance claims data to evaluate the practice patterns of timing and modality for hydroxychloroquine retinopathy screening in South Korea.

## Introduction

Hydroxychloroquine is a 4-aminoquinoline derivative that is widely used for the treatment of several rheumatologic and dermatologic disorders, such as systemic lupus erythematosus and rheumatoid arthritis.^1,2,3^ It is known to be more effective and safer than its parent drug (chloroquine)^4,5^; however, retinal toxicity, a well-known adverse effect of the drug, is a significant concern for both prescribing physicians and ophthalmologists.

Because of irreversible and progressive vision loss caused by retinal toxic effects potentially leading to blindness, careful attention and screening are required for long-term hydroxychloroquine users.^6,7^ The American Academy of Ophthalmology (AAO) provided recommendations for screening in 2002,^8^ with subsequent revisions in 2011 and 2016.^6,9^ The most recent guideline suggests spectral-domain optical coherence tomography (SD-OCT), fundus autofluorescence (FAF), automated visual field (VF), and multifocal electroretinography (mfERG) as recommended tests, with at least 1 objective abnormal test result confirming subjective abnormality suggested for the diagnosis of retinopathy.^6^ For timing of screening, all patients beginning long-term hydroxychloroquine therapy should have a baseline ophthalmologic examination within the first year of use, whereas annual monitoring should begin after 5 years of use or sooner for long-term users. Recently, the Royal College of Ophthalmologists (RCOphth) published national guidelines in the UK suggesting the above 4 tests for screening; however, the primary test recommendation was SD-OCT and wide-field FAF instead of the AAO’s recommended SD-OCT and automated VF.^6,10^

Because not all recommended tests may be available in ophthalmic practice, the modalities used for retinopathy screening may vary nationally, regionally, or even according to the level of health care. Therefore, despite the dissemination of national guidelines, real-world screening practices sometimes differ widely. In a large, multispecialty ophthalmic practice in the US, only approximately half of patients underwent appropriate screening according to the 2011 AAO guideline, and the most preferred modalities were SD-OCT and 10-2 Humphrey visual field testing.^11^ Although a few reports on the practice patterns for hydroxychloroquine retinopathy screening have been published,^11,12,13^ to our knowledge, the trends or changes in screening practice patterns over time have not yet been reported. Furthermore, nationwide patterns across diverse ophthalmic practices in South Korea have also not been investigated.

Because South Korea has a mandatory universal health insurance system that provides medical care to almost its entire population, its data on health claims may be ideal for investigation of national practice patterns. The objective of this study was to evaluate the annual trends of ophthalmic practice patterns for timing and modalities of hydroxychloroquine retinopathy screening between January 1, 2015, and December 31, 2021, in South Korea and their adherence to the current guidelines.

## Methods

### Study Populations

The Health Insurance Review and Assessment (HIRA) database includes information on diagnoses, procedures (examinations), prescription records, visit dates, and demographic characteristics of approximately 50 million people. It also contains all health care use information for inpatient and outpatient visits using codes from the *Korean Standard Classification of Diseases*, *8th Revision*, with a few modifications based on the *International Statistical Classification of Diseases and Related Health Problems, Tenth Revision* (*ICD-10*). The current nationwide population-based study identified patients at risk of hydroxychloroquine retinopathy using health claims data recorded in the HIRA database between January 1, 2007, and December 31, 2021.

Patients at risk were identified as those treated with hydroxychloroquine between January 1, 2009, and December 31, 2020, for 6 months or more because there are very few cases in the literature of hydroxychloroquine retinopathy having developed earlier than 6 months from the initiation of hydroxychloroquine treatment.^14,15^ Among this population, those who used hydroxychloroquine before January 1, 2009, were excluded because their therapy had been initiated prior to the data inclusion period, and thus, the duration of their treatment could not be accurately assessed. We assumed that if patients did not use hydroxychloroquine between January 1, 2007, and December 31, 2008, but used it after January 1, 2009, they were hydroxychloroquine initiators after 2009. Furthermore, those who underwent one of 4 standard screening tests (ie, SD-OCT, FAF, VF, and mfERG) for any ophthalmic disease (*ICD-10* codes H00-H59) prior to the initial use of hydroxychloroquine were excluded to remove patient visits scheduled for monitoring of preexisting ophthalmic diseases. All of the inclusion and exclusion criteria as well as the number of patients are detailed in the eFigure in Supplement 1. This nationwide, population-based retrospective cohort study was approved by the institutional review board of Hanyang University Hospital and was conducted in accordance with the tenets of the Declaration of Helsinki.^16^ The need for informed consent was waived by the institutional review board owing to the retrospective nature of this study and use of a deidentified data set. This report followed the Strengthening the Reporting of Observational Studies in Epidemiology (STROBE) reporting guideline for cohort studies.

### Definitions

Several definitions were used in this study. *Users at risk* were defined as patients taking hydroxychloroquine for 6 months or more, and *long-term users* were defined as patients taking hydroxychloroquine for 5 years or more as measured in total prescription days. *Baseline test* was defined as the first ophthalmic examination using fundoscopy or fundus photography or any of the 4 AAO-recommended modalities (SD-OCT, FAF, VF, and mfERG) performed for those at risk since hydroxychloroquine treatment was initiated. However, we did not consider the first ophthalmic examination performed after 5 years of hydroxychloroquine use as a baseline test, as this should be performed for regular (annual) monitoring according to the AAO guidelines. *Monitoring examinations* were defined as tests performed using any of the 4 recommended modalities after 5 years of hydroxychloroquine use.

According to the most recent AAO and RCOphth guidelines, respectively, “at least 1 objective test abnormality confirming subjective abnormality” and “2 test abnormalities” are required for diagnosis of hydroxychloroquine retinopathy.^6,10^ Correspondingly, both national guidelines imply that at least 2 tests need to be performed for screening and that 2 test abnormalities are required for diagnosis. Accordingly, adherence of monitoring examinations to the current national guidelines was assessed as appropriate (annual screening using ≥2 tests among the 4 recommended tests), unscreened (no examination using any of the 4 screening tests), or underscreened (insufficient number of tests) for each long-term user. Baseline screening was considered appropriate if a patient at risk had undergone fundoscopy or fundus photography within 1 year.^6^

### Statistical Analysis

The HIRA database was used in the analysis of patients treated with hydroxychloroquine, considering variables such as personal identification, drug component code, treatment date, treatment code, and total number of prescription days. However, in January 2015, health claims data on SD-OCT became available in the HIRA database. Thus, the performance of tests and list of tests performed for baseline and monitoring examinations could be assessed correctly from 2015 onward. Consequently, our analyses incorporated data from baseline screening and monitoring examinations obtained from 2015.

Categorical variables were recorded as frequency and percentage and continuous variables as mean (SD) or median (IQR) values. The χ^2^ test was used to compare categorical variables between groups. All *P* values were from 2-sided tests, and results were deemed statistically significant at *P* < .05. SAS Enterprise Guide Software, version 7.1 (SAS Institute Inc) was used for all analyses.

## Results

### Population of Hydroxychloroquine Users and Trends Over Time

Our study included 65 406 users at risk of retinopathy (mean [SD] age, 53.0 [15.5] years; 50 622 women [77.4%]); 29 776 were long-term users (mean [SD] age, 50.1 [14.7] years; 24 898 women [83.6%]) (Table 1). eTable 1 in Supplement 1 shows the annual numbers of hydroxychloroquine users and the yearly numbers of hydroxychloroquine initiators at risk of retinopathy in South Korea between 2009 and 2020. Over this period, the number of initiators gradually decreased from 0.031% in 2009 to 0.017% in 2020, indicating a decreasing trend in hydroxychloroquine initiators, particularly those with rheumatoid arthritis (eTable 2 in Supplement 1). However, the annual number of hydroxychloroquine users increased from 108 660 in 2009 to 124 660 in 2020, as shown in eTable 1 in Supplement 1. Accordingly, the percentage of hydroxychloroquine users among the entire Korean population (approximately 50 million) slightly increased from 0.22% in 2009 to 0.24% in 2020.

**Table 1.** Demographic and Clinical Information on Hydroxychloroquine Users

Characteristic	No. (%)	
	Overall users at risk (N = 65 406)	Long-term users (n = 29 776)
Sex		
Male	14 784 (22.6)	4878 (16.4)
Female	50 622 (77.4)	24 898 (83.6)
Age, mean (SD), y	53.0 (15.5)	50.1 (14.7)
Age groups, y		
<20	1581 (2.4)	941 (3.2)
20-29	3860 (5.9)	2029 (6.8)
30-39	7065 (10.8)	3932 (13.2)
40-49	12 149 (18.6)	6395 (21.5)
50-59	18 022 (27.6)	8404 (28.2)
60-69	13 328 (20.4)	5406 (18.2)
≥70	9401 (14.4)	2669 (9.0)
Comorbidities (at baseline)		
Diabetes	15 275 (23.4)	4664 (15.7)
Hypertension	24 262 (37.1)	10 690 (35.9)
Medical specialties prescribing hydroxychloroquine		
Rheumatology	36 484 (55.8)	16 812 (56.5)
Internal medicine other than rheumatology	21 130 (32.3)	9576 (32.2)
Others	7792 (11.9)	3388 (11.4)
Diagnosis requiring hydroxychloroquine use		
SLE	9823 (15.0)	5288 (17.8)
RA	44 553 (68.1)	19 384 (65.1)
Others	11 030 (16.9)	5104 (17.1)
Duration of hydroxychloroquine use, mean (SD), mo	28.7 (19.6)	92.2 (21.1)

Abbreviations: RA, rheumatoid arthritis; SLE, systemic lupus erythematosus.

### Performance, Timing, and Modalities of Baseline Examinations

The frequency of patients undergoing baseline examinations and the timing and modalities of screening examinations are presented in Table 2. The number of patients who underwent baseline examinations within 1 year after initiation of hydroxychloroquine therapy was 13 597 and within 5 years after initiation of hydroxychloroquine therapy was 28 994, representing 20.8% and 44.3%, respectively, of the patients at risk and indicating that 79.2% had not undergone baseline screening within 1 year and 55.7% had not undergone baseline screening within 5 years. The Figure shows the yearly trend of baseline examinations (performed within 1 year of hydroxychloroquine use), demonstrating a gradual increase from 16.6% in 2015 to 25.6% in 2021. The median interval from hydroxychloroquine initiation to the first ophthalmic (baseline) examination was 405 days (IQR, 119-859 days).

**Table 2.** Descriptive Statistics of Timing and Modalities Used for Baseline Examination

Characteristic	Value
Timing of baseline examination	
No. of patients receiving baseline examination within 5 y of hydroxychloroquine use/No. of patients at risk (%)	28 994/65 406 (44.3)
No. of patients receiving baseline examination within 1 y of hydroxychloroquine use/No. of patients at risk (%)	13 597/65 406 (20.8)
Timing of baseline examination since hydroxychloroquine use, median (IQR), d	405 d (119-859)
Timing of annual monitoring	
No. of patients receiving any monitoring examination after year 5/No. of long-term users (%)	9406/29 776 (31.6)
No. of patients receiving monitoring examination in year 5/No. of long-term users (%)^a^	4028/29 776 (13.5)
No. of monitoring examinations per year after 5 y of hydroxychloroquine use (SD), No./y	0.6 (0.3)
Mean (SD)/median (IQR) timing of the 1st monitoring examination since hydroxychloroquine use, mo	65.5 (3.6)/65.3 (62.4-68.6)
Mean (SD)/median (IQR) interval of monitoring between the 1st and 2nd examinations, mo	14.9 (10.2)/17.3 (12.4-27.6)
Mean (SD)/median (IQR) interval of monitoring between the 2nd and 3rd examinations, mo	14.3 (6.6)/12.4 (11.6-15.6)
Modalities used for screening	
Fundoscopy or fundus photography, baseline, No. (%)	12 904 (94.9)
Optical coherence tomography, baseline/monitoring, No. (%)	5427 (39.9)/12 337 (84.0)
Automated visual fields, baseline/monitoring, No. (%)	3443 (25.3)/6214 (42.3)
Fundus autofluorescence, baseline/monitoring, No. (%)	1928 (14.2)/3535 (24.1)
Multifocal electroretinogram, baseline/monitoring, No. (%)	111 (0.8)/138 (0.9)

^a^ Between day 1825 (day 0 of year 5) and 2189 (day 364 of year 5) of hydroxychloroquine use.

**Figure.**
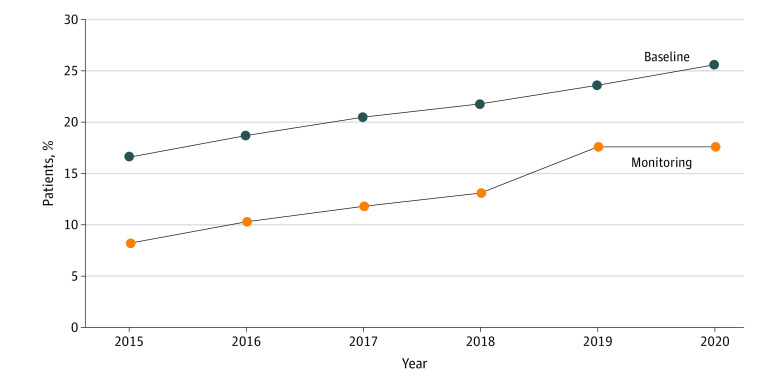
Percentage of Patients Receiving Baseline Screening Within 1 Year of Hydroxychloroquine Use and Annual Monitoring in Year 5 in Each Year Between 2015 and 2020

Baseline screening was performed most commonly using fundus examination (94.9%) either by fundoscopy or fundus photography, followed by OCT (39.9%) and VF (25.3%). The combinations of tests used for baseline screening are detailed in eTable 3 in Supplement 1.

### Performance, Timing, and Modalities of Regular Monitoring Among Long-term Users

Detailed statistics on the timing and modalities used for regular monitoring among long-term users are presented in Table 2. Among the 29 776 long-term users, 4028 (13.5%) underwent ophthalmic examination using at least 1 of the 4 recommended modalities (SD-OCT, VF, FAF, and mfERG) in year 5 at 1825 to 2189 days of hydroxychloroquine use. The percentage of patients undergoing any monitoring examination after 5 years (1825 days) of hydroxychloroquine use among the long-term users was 31.6%, indicating that 68.4% had not undergone monitoring screening after 5 years. Among those who had undergone any monitoring examination, the mean (SD) time of the first monitoring examination was 65.5 (3.6) months and the median time of the first monitoring examination was 65.3 months (IQR, 62.4-68.6 months). The timing of subsequent monitoring examinations also is presented in Table 2, showing a trend toward shorter intervals between later examinations. Among the modalities used for retinopathy screening, SD-OCT was the most commonly used modality, as 84.0% of patients underwent that test, whereas 42.3% underwent VF, 24.1% underwent FAF, and 0.9% underwent mfERG. The combinations of tests used for regular monitoring are detailed in eTable 4 in Supplement 1, demonstrating that OCT only; OCT and VF; and OCT, VF, and FAF, in order, were the most common modalities used for monitoring.

### Adherence of Baseline Screening and Regular Monitoring Practices to Current AAO Guidelines

The trends in the proportions of patients undergoing baseline examination by fundoscopy or fundus photography within 1 year of hydroxychloroquine use are summarized in eTable 5 in Supplement 1. The yearly cohort of hydroxychloroquine initiators from 2015 to 2020 showed a gradual increase (from 15.2% to 24.6%) in the percentage of patients undergoing baseline fundus examinations within 1 year and (thereby) adhering to the 2016 AAO guideline’s recommendation for baseline examinations. Among the patients who used hydroxychloroquine for 5 years, analysis of the proportion who underwent monitoring examinations in year 5 also revealed a gradual increase in the screened population from 8.2% in 2015 to 17.6% in 2020 (Figure). Specifically, both the appropriately screened and underscreened populations increased significantly from 3.9% in 2015 to 9.1% in 2020 and from 4.3% to 8.5%, respectively (Table 3).

**Table 3.** Proportion of Long-term Users Receiving a Monitoring Examination in Year 5

Year	Adherence to the guideline for regular monitoring, No./total No. (%)			
	Screened			Unscreened
	Appropriate	Underscreened	Total	
2015	162/4165 (3.9)	178/4165 (4.3)	340/4165 (8.2)	3825/4165 (91.8)
2016	213/4473 (4.8)	248/4473 (5.5)	461/4473 (10.3)	4012/4473 (89.7)
2017	291/4951 (5.9)	294/4951 (5.9)	585/4951 (11.8)	4366/4951 (88.2)
2018	297/4845 (6.1)	336/4845 (6.9)	633/4845 (13.1)	4212/4845 (86.9)
2019	443/4845 (9.1)	410/4845 (8.5)	853/4845 (17.6)	3992/4845 (82.4)
2020	401/4408 (9.1)	374/4408 (8.5)	775/4408 (17.6)	3633/4408 (82.4)
2021^a^	186/2089 (8.9)	195/2089 (9.3)	381/2089 (18.2)	1708/2089 (81.8)

^a^ The mean (SD) observation period until the study end date (ie, December 31, 2021) was 145.9 (96.8) days for 2021.

The history of baseline examination was significantly associated with monitoring examinations in the cohort of long-term users (Table 4). For example, the percentage of patients undergoing any monitoring examination in year 5 was 2.3 times greater for those who had received baseline screening than for those who had not (27.4% vs 11.9%; *P* < .001). eTable 6 in Supplement 1 indicates the association of medical specialties prescribing hydroxychloroquine and indications of hydroxychloroquine use with baseline screening and regular monitoring practices.

**Table 4.** Association Between Performances in Baseline and Monitoring in Year 5 Examinations

Baseline examination	No./total No. (%)		*P* value
	Monitoring performed	Monitoring not performed	
Not performed	3156/26 588 (11.9)	23 432/26 588 (88.1)	<.001
Performed	872/3188 (27.4)	2316/3188 (72.7)	

## Discussion

The present study revealed a trend in nationwide use of hydroxychloroquine by patients at risk of retinopathy in South Korea. Although adherence to the current AAO guidelines regarding the timing and modalities used for both baseline and monitoring examinations improved over time, most long-term hydroxychloroquine users remained unscreened after 5 years of hydroxychloroquine use.

Our findings on the use of hydroxychloroquine revealed that during the study period, the annual number of new hydroxychloroquine users at risk of retinopathy decreased, although the annual number of overall hydroxychloroquine users in South Korea increased. Accordingly, more patients should be considered for screening by ophthalmologists, although over time, the target population for baseline screening has decreased every year. To our knowledge, this is the first report on the use of hydroxychloroquine at a national level that also estimates the proportion of hydroxychloroquine users (0.21%-0.24%) and initiators (0.017%-0.031%) among the entire national population. These numbers and percentages of individual drug users, being not negligible, raise public health concerns urging the establishment and implementation of screening programs for retinopathy.

The AAO recommendations published in 2011 and 2016 defined risk factors for hydroxychloroquine retinopathy, provided dosage recommendations, and suggested when and how to perform baseline and annual screenings.^6,9^ The timing and modalities used for screening are crucial for timely detection of hydroxychloroquine retinopathy and minimization of the risk of irreversible vision loss due to retinal toxicity.^6,17^ However, our findings on timing and modalities suggest that most patients remained unscreened by the baseline (79.2% within 1 year and 55.7% within 5 years) or monitoring (68.4% after 5 years) examination. Unscreened long-term users were of particular concern, as this situation could lead to detection of retinopathy at the symptomatic late stage.^6^ Underscreened patients for whom an insufficient number of tests had been performed were not as remarkable as unscreened patients, and the screening modalities mostly included SD-OCT and VF, in agreement with the AAO guideline on suggested primary tests.^6^ Therefore, our results suggest that performance of screening itself, rather than how it is performed, may be more important for minimization of delayed diagnosis and associated vision loss in real-world practices of hydroxychloroquine retinopathy screening. Of highest importance, patients taking hydroxychloroquine for diverse indications should be referred by prescribing physicians to ophthalmologists to undergo screening examinations.

Our study showed a significant association between baseline and monitoring examinations. The percentage of patients undergoing monitoring examinations in year 5 was 2.3 times greater in those who had been screened with baseline examinations than in those who had not (27.4% vs 11.9%). The patients who had received baseline screening might have received annual screening by the same physicians who knew and adhered to the guidelines well, or the indication of baseline screening in the medical record might have led to continued annual screening by the same or other physicians. Baseline screening was performed mainly to evaluate any preexisting ocular condition and to document fundus appearance and functional status as unaffected by hydroxychloroquine.^6,17^ The AAO guideline indicates that the baseline examination may also provide an opportunity to advise patients about regular screening.^6^ Our results on the significant association between baseline and monitoring examinations also support this supplementary but important role of baseline screening. Accordingly, another objective of screening should be patient education, which could significantly affect future visits by motivating and educating patients to undergo regular monitoring.

Despite the poor adherence to the current guideline that we observed in our results, the changes in screening practices between 2015 and 2020 (Figure) can be considered to be significant because the proportion of screened patients in year 5 increased from 8.2% to 17.6% during the 6-year period and the proportion of appropriately screened patients more than doubled from 3.9% to 9.1%. These changes could have been due to better awareness among ophthalmologists regarding timing and modalities for appropriate screening of hydroxychloroquine retinopathy. Although no national guideline for hydroxychloroquine retinopathy has yet been established in Korea, to our knowledge, the most recent AAO guideline published in 2016 might have played a significant role in enhancing ophthalmologists’ awareness of screening examinations worldwide and, thus, influencing the percentage of screened patients during this period.

Another important issue in hydroxychloroquine retinopathy is late diagnosis, which is more frequent among Asian populations than other ethnic populations.^6,17,18^ Ethnic differences in retinopathy patterns, especially the more peripheral distribution of retinal damage among Asian patients,^18,19^ has been considered a possible cause of this occurrence because late diagnosis is more common in pericentral retinopathy.^17,18^ Several reports from Korea have shown that approximately half of patients had severe disease at the time of diagnosis.^20,21,22,23^ Peripheral damage undetected by conventional imaging modalities may also partly explain late diagnoses among the Korean population^17,24^; however, our results also suggest that unscreened long-term hydroxychloroquine users, which comprise over 60% of long-term users, also account for late diagnosis among Korean patients. Accordingly, screening practices should be investigated carefully, particularly regarding whether the timing and modalities used for screening are appropriately in accordance with established guidelines, to elucidate the causes of late diagnosis of hydroxychloroquine retinopathy in any population.

### Limitations

Our study has several limitations requiring careful consideration. We could not exclude ophthalmic examinations performed on participants for purposes other than toxicity screening. To minimize the number of examinations for other purposes (eg, diabetic retinopathy screening), we excluded patients with any ophthalmic disease who had undergone any of the 4 recommended tests prior to hydroxychloroquine initiation. Furthermore, we have confirmed that the data on baseline examinations (eTable 7 in Supplement 1) and monitoring examinations (eTable 8 in Supplement 1) were comparable even after excluding patients with common ophthalmologic diseases and those with diabetes. However, although the tests were performed for other purposes, the same tests recommended for hydroxychloroquine retinopathy screening may also reveal structural or functional abnormalities. This ophthalmic practice may lead to diagnosis of hydroxychloroquine retinopathy based on the characteristic structural and functional defects in the parafoveal or pericentral areas, on which screening practices also focus. In addition, we defined patients at risk as those treated with hydroxychloroquine for 6 months or more; however, no consensus on patients at risk requiring screening examinations currently exists. Daily dose, or dose divided by body weight, should be carefully considered together with duration of hydroxychloroquine use to define the risk of retinopathy,^25^ but this information was not available to the present study. Use of typical hydroxychloroquine doses for a few months does entail a very low risk of retinopathy; thus, retinopathy screening is considered unnecessary for such short-term users.^14^ However, in a trial of high-dose hydroxychloroquine for cancer, one patient developed subtle changes that were detected on OCT after 11 months.^15^ Without information on hydroxychloroquine dose, we used the criteria of hydroxychloroquine use for 6 months or more to include patients at risk of retinopathy who had been receiving diverse hydroxychloroquine doses for different indications. Finally, test quality could not be assessed in our study; for example, a test performed using time-domain OCT could not be discriminated from one performed using SD-OCT because the same health claim code for OCT is used for the different devices. Therefore, even though a quality-assured examination is a prerequisite for assessing adherence to AAO guidelines, we could not evaluate the quality of the screening service in this study.

## Conclusions

The present study assessed the timing and modalities of screening for hydroxychloroquine retinopathy at a national level in South Korea between January 1, 2015, and December 31, 2021. Unscreened long-term users are of major concern with regard to screening practices for hydroxychloroquine retinopathy, and further measures or national guidelines are essential to facilitate patient visits to ophthalmologists for retinopathy screening. The significant association between baseline and monitoring examinations highlights the importance of a baseline examination, which promotes subsequent follow-up and reduces the number of unscreened long-term users.

## Notes

Supplement 1. **eFigure.** A Flowchart of the Study Population and Inclusion/Exclusion Criteria Used in This Study **eTable 1.** Annual Number of Patients Using Hydroxychloroquine, Proportion in Entire Korean Population in Each Year, and Yearly Number of Patients at Risk (Duration of Hydroxychloroquine Use ≥ 6 Months) Who Initiated Hydroxychloroquine Therapy Between 2009 and 2020 **eTable 2.** Annual Number of Patients Who Initiated Hydroxychloroquine Therapy Between 2009 and 2020 According to Indications of Use **eTable 3.** Combinations of Tests Used for Baseline Examinations **eTable 4.** Combinations of 4 Recommended Screening Tests Used for Regular Monitoring **eTable 5.** Analyses of Proportion of Patients Receiving Baseline Examinations With Fundus Examination Within One Year of Hydroxychloroquine Use **eTable 6.** Association of Department of Prescription or Indications of Hydroxychloroquine Use With Performance of Baseline (Within 1 Year) and Monitoring (After 5 Years) Examinations **eTable 7.** Descriptive Statistics of Timing and Modalities Used for Baseline Examination Among Overall Patients at Risk (n = 65,406), Those Without Common Ophthalmologic Diseases (n = 61,841), and Those Without Diabetes Mellitus (DM; n = 51,548) Between 2015 and 2020 **eTable 8.** Descriptive Statistics of Timing and Modalities Used for Regular Monitoring Among Long-Term Users Without Common Ophthalmologic Diseases (n = 23,534) and Those Without Diabetes Mellitus (DM; n = 18,513) Between 2015 and 2021

Supplement 2.
Data Sharing Statement
