# Supplementary material for: Practice Patterns of Screening for Hydroxychloroquine Retinopathy in South Korea
Source: JAMA Netw Open. 2023 May 23;6(5):e2314816. doi: 10.1001/jamanetworkopen.2023.14816 (PMC10208143; doi:10.1001/jamanetworkopen.2023.14816)
Supplement: Supplement 1. — eFigure. A Flowchart of the Study Population and Inclusion/Exclusion Criteria Used in This Study eTable 1. Annual Number of Patients Using Hydroxychloroquine, Proportion in Entire Korean Population in Each Year, and Yearly Number of Patients at Risk (Duration of Hydroxychloroquine Use ≥ 6 Months) Who Initiated Hydroxychloroquine Therapy Between 2009 and 2020 eTable 2. Annual Number of Patients Who Initiated Hydroxychloroquine Therapy Between 2009 and 2020 According to Indications of Use eTable 3. Combinations of Tests Used for Baseline Examinations eTable 4. Combinations of 4 Recommended Screening Tests Used for Regular Monitoring eTable 5. Analyses of Proportion of Patients Receiving Baseline Examinations With Fundus Examination Within One Year of Hydroxychloroquine Use eTable 6. Association of Department of Prescription or Indications of Hydroxychloroquine Use With Performance of Baseline (Within 1 Year) and Monitoring (After 5 Years) Examinations eTable 7. Descriptive Statistics of Timing and Modalities Used for Baseline Examination Among Overall Patients at Risk (n = 65,406), Those Without Common Ophthalmologic Diseases (n = 61,841), and Those Without Diabetes Mellitus (DM; n = 51,548) Between 2015 and 2020 eTable 8. Descriptive Statistics of Timing and Modalities Used for Regular Monitoring Among Long-Term Users Without Common Ophthalmologic Diseases (n = 23,534) and Those Without Diabetes Mellitus (DM; n = 18,513) Between 2015 and 2021 [file jamanetwopen-e2314816-s001.pdf]

Supplementary Online Content

Kim J, Kim KE, Kim JH, Ahn SJ. Practice patterns of screening for hydroxychloroquine retinopathy in South Korea. *JAMA Netw Open.* 2023;6(5):e2314816. doi:10.1001/jamanetworkopen.2023.14816

eFigure. A Flowchart of the Study Population and Inclusion/Exclusion Criteria Used in This Study
eTable 1. Annual Number of Patients Using Hydroxychloroquine, Proportion in Entire Korean Population in Each Year, and Yearly Number of Patients at Risk (Duration of Hydroxychloroquine Use ≥ 6 Months) Who Initiated Hydroxychloroquine Therapy Between 2009 and 2020
eTable 2. Annual Number of Patients Who Initiated Hydroxychloroquine Therapy Between 2009 and 2020 According to Indications of Use
eTable 3. Combinations of Tests Used for Baseline Examinations
eTable 4. Combinations of 4 Recommended Screening Tests Used for Regular Monitoring
eTable 5. Analyses of Proportion of Patients Receiving Baseline Examinations With Fundus Examination Within One Year of Hydroxychloroquine Use
eTable 6. Association of Department of Prescription or Indications of Hydroxychloroquine Use With Performance of Baseline (Within 1 Year) and Monitoring (After 5 Years) Examinations
eTable 7. Descriptive Statistics of Timing and Modalities Used for Baseline Examination Among Overall Patients at Risk (n = 65,406), Those Without Common Ophthalmologic Diseases (n = 61,841), and Those Without Diabetes Mellitus (DM; n = 51,548) Between 2015 and 2020
eTable 8. Descriptive Statistics of Timing and Modalities Used for Regular Monitoring Among Long-Term Users Without Common Ophthalmologic Diseases (n = 23,534) and Those Without Diabetes Mellitus (DM; n = 18,513) Between 2015 and 2021

This supplementary material has been provided by the authors to give readers additional information about their work.

© 2023 Kim J et al. *JAMA Network Open.*

eFigure. A Flowchart of the Study Population and Inclusion/Exclusion Criteria Used in This Study.

HCQ = hydroxychloroquine

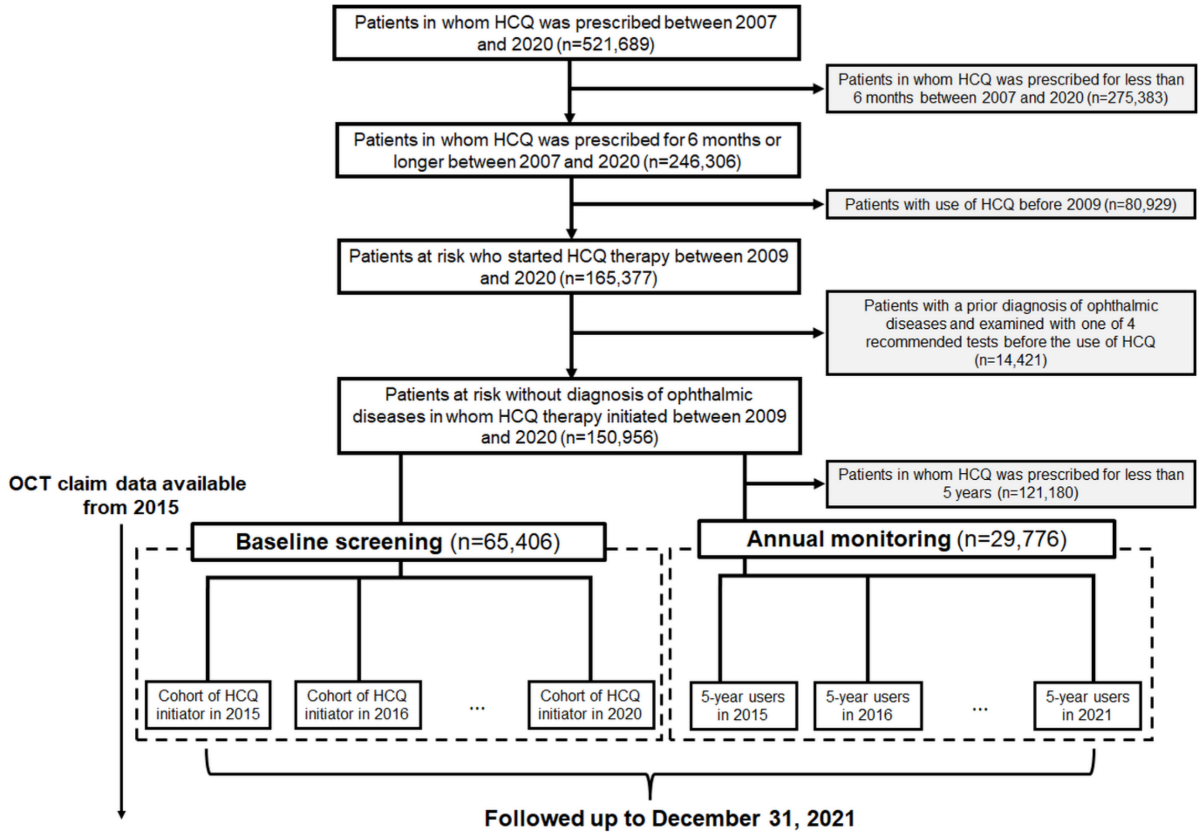

© 2023 Kim J et al. *JAMA Network Open.*

eTable 1. Annual Number of Patients Using Hydroxychloroquine, Proportion in Entire Korean Population in Each Year, and Yearly Number of Patients at Risk (Duration of Hydroxychloroquine Use ≥ 6 Months) Who Initiated Hydroxychloroquine Therapy Between 2009 and 2020

Year	Total number of patients using hydroxychloroquine (% among entire Korean population in each year†)	Annual number of patients at risk who initiated hydroxychloroquine therapy (% among Korean population†)
2009	108,660 (0.22%)	15,387 (0.031%)
2010	104,525 (0.21%)	14,744 (0.030%)
2011	105,825 (0.21%)	13,722 (0.027%)
2012	111,461 (0.22%)	14,726 (0.029%)
2013	114,487 (0.22%)	13,910 (0.028%)
2014	116,487 (0.23%)	13,061 (0.026%)
2015	117,336 (0.23%)	12,425 (0.024%)
2016	119,924 (0.23%)	12,106 (0.024%)
2017	120,387 (0.23%)	11,232 (0.022%)
2018	121,459 (0.23%)	10,781 (0.021%)
2019	121,923 (0.24%)	9,943 (0.019%)
2020	124,660 (0.24%)	8,919 (0.017%)

†Obtained by dividing the number of hydroxychloroquine users by that of the entire Korean population in each year (from 49,307,835 in 2009 to 51,836,239 in 2020)

© 2023 Kim J et al. *JAMA Network Open.*

eTable 2. Annual Number of Patients Who Initiated Hydroxychloroquine Therapy Between 2009 and 2020 According to Indications of Use

Year	Annual number of patients at risk who initiated hydroxychloroquine therapy (%)		
	SLE	RA	Others
2009	1241 (8.1%)	11025 (71.7%)	3121 (20.3%)
2010	1218 (8.3%)	10438 (70.8%)	3088 (20.9%)
2011	1374 (10.0%)	9399 (68.5%)	2949 (21.5%)
2012	1477 (10.0%)	10751 (73.0%)	2498 (17.0%)
2013	1489 (10.7%)	10155 (73.0%)	2266 (16.3%)
2014	1585 (12.1%)	9442 (72.3%)	2034 (15.6%)
2015	1610 (13.0%)	8773 (70.6%)	2042 (16.4%)
2016	1740 (14.4%)	8223 (67.9%)	2143 (17.7%)
2017	1650 (14.7%)	7676 (68.3%)	1906 (17.0%)
2018	1671 (15.5%)	7339 (68.1%)	1771 (16.4%)
2019	1550 (15.6%)	6683 (67.2%)	1710 (17.2%)
2020	1602 (18.0%)	5859 (65.7%)	1458 (16.4%)

SLE = systemic lupus erythematosus; RA = rheumatoid arthritis

© 2023 Kim J et al. *JAMA Network Open.*

eTable 3. Combinations of Tests Used for Baseline Examinations

Tests performed	N (%)
Fundus examination only	6,963 (51.2%)
Fundus examination + OCT	2,396 (17.6%)
Fundus examination + OCT + VF	1,068 (7.9%)
Fundus examination + OCT + VF + FAF	1,033 (7.6%)
Fundus examination + OCT + VF + FAF + mfERG	43 (0.3%)
Fundus examination + OCT + VF + mfERG	20 (0.2%)
Fundus examination + OCT + FAF	372 (2.7%)
Fundus examination + OCT + FAF + mfERG	7 (0.05%)
Fundus examination + OCT + mfERG	11 (0.08%)
Fundus examination + VF	666 (5.0%)
Fundus examination + VF + FAF	190 (1.4%)
Fundus examination + VF + FAF + mfERG	10 (0.07%)
Fundus examination + VF + mfERG	8 (0.06%)
Fundus examination + FAF	110 (0.8%)
Fundus examination + FAF + mfERG	2 (0.01%)
Fundus examination + mfERG	5 (0.04%)
OCT only	272 (2.0%)
OCT + VF	116 (0.9%)
OCT + VF + FAF	73 (0.5%)
OCT + VF + FAF + mfERG	1 (0.01%)
OCT + VF + mfERG	3 (0.02%)
OCT + FAF	12 (0.09%)
VF only	140 (1.0%)
VF + FAF	72 (0.5%)
FAF only	3 (0.02%)
mfERG only	1 (0.01%)

FAF, fundus autofluorescence; mfERG, multifocal electroretinogram; OCT, optical coherence tomography; VF, visual fields

© 2023 Kim J et al. *JAMA Network Open.*

eTable 4. Combinations of 4 Recommended Screening Tests Used for Regular Monitoring

Tests performed	N (%)
OCT only	7,128 (48.6%)
OCT + VF	2,254 (15.4%)
OCT + VF + FAF	1,829 (12.5%)
OCT + VF + FAF + mfERG	37 (0.3%)
OCT + VF + mfERG	22 (0.2%)
OCT + FAF	1,047 (7.1%)
OCT + FAF + mfERG	9 (0.06%)
OCT + mfERG	11 (0.07%)
VF only	1,686 (11.5%)
VF + FAF	354 (2.4%)
VF + FAF + mfERG	10 (0.07%)
VF + mfERG	22 (0.2%)
FAF only	244 (1.7%)
FAF + mfERG	5 (0.03%)
mfERG only	22 (0.2%)

FAF, fundus autofluorescence; mfERG, multifocal electroretinogram; OCT, optical coherence tomography; VF, visual field.

© 2023 Kim J et al. *JAMA Network Open.*

eTable 5. Analyses of Proportion of Patients Receiving Baseline Examinations With Fundus Examination Within One Year of Hydroxychloroquine Use

Year of initial hydroxychloroquine use	Adherence to the guideline for baseline examination
	Test performed/total number of patients (%)
2015	1,891/12,425 (15.2%)
2016	2,131/12,106 (17.6%)
2017	2,185/11,232 (19.5%)
2018	2,244/10,781 (20.8%)
2019	2,256/9,943 (22.7%)
2020	2,197/8,919 (24.6%)

© 2023 Kim J et al. *JAMA Network Open.*

eTable 6. Association of Department of Prescription or Indications of Hydroxychloroquine Use With Performance of Baseline (Within 1 Year) and Monitoring (After 5 Years) Examinations

**Characteristics**	**Baseline exam** Performed/total (%)	**p-value**	**Monitoring** Performed/total (%)	**p-value**
**Medical specialties prescribing HCQ**				
Rheumatology	8,353/36,484 (22.9%)		5,884/16,812 (35.0%)	
Internal medicine other than rheumatology	3,625/21,130 (17.2%)	<.001	2,578/9,576 (26.9%)	<.001
Others	1,619/7,792 (20.8%)		944/3,388 (27.9%)	
**Diagnosis for HCQ therapy**				
SLE	2,940/9,823 (29.9%)		2,240/5,288 (42.4%)	
RA	8,032/44,553 (18.0%)	<.001	5,273/19,384 (27.2%)	<.001
Others	2,625/11,030 (23.8%)		1,893/5,104 (37.1%)	

HCQ = hydroxychloroquine; SLE = systemic lupus erythematosus; RA = rheumatoid arthritis

© 2023 Kim J et al. *JAMA Network Open.*

eTable 7. Descriptive Statistics of Timing and Modalities Used for Baseline Examination Among Overall Patients at Risk (n = 65,406), Those Without Common Ophthalmologic Diseases (n = 61,841), and Those Without Diabetes Mellitus (DM; n = 51,548) Between 2015 and 2020

Characteristics	Overall	Without common ophthalmologic diseases*	Without DM
No. of patients receiving baseline examination within 5 years of hydroxychloroquine use/No. of patients at risk (%)	28,994 (44.3%)	25,923 (41.9%)	21,757 (42.2%)
No. of patients receiving baseline examination within 1 year of hydroxychloroquine use/No. of patients at risk (%)	13,597 (20.8%)	10,993 (17.8%)	9,820 (19.1%)
Timing of the baseline examination since hydroxychloroquine use, median (IQR)	405 days (119–859 days)	460 days (114–911 days)	429 days (126–896 days)
Modalities used for baseline examination (%)			
Fundoscopy/fundus photography	12,904 (94.9%)	10,434 (94.9%)	9,270 (94.4%)
Optical coherence tomography	5,427 (39.9%)	3,868 (35.2%)	3,995 (40.7%)
Automated visual fields	3,443 (25.3%)	2,733 (24.9%)	2,814 (28.7%)
Fundus autofluorescence	1,928 (14.2%)	1,542 (14.0%)	1,585 (16.1%)
Multifocal electroretinogram	111 (0.8%)	88 (0.8%)	92 (0.9%)

*These include glaucoma, common macular diseases (e.g. age-related macular degeneration, macular edema, and central serous chorioretinopathy), and diabetic retinopathy.

© 2023 Kim J et al. *JAMA Network Open.*

eTable 8. Descriptive Statistics of Timing and Modalities Used for Regular Monitoring Among Long-Term Users Without Common Ophthalmologic Diseases (n = 23,534) and Those Without Diabetes Mellitus (DM; n = 18,513) Between 2015 and 2021

Characteristics	Without common ophthalmologic diseases	Without DM*
No. of patients receiving any monitoring examination after year 5/No. of long-term users (%)	5,079/23,534 (21.6%)	5,468/18,513 (29.5%)
No. of patients receiving monitoring examination in year 5/No. of long-term users (%)	2,041/23,534 (8.7%)	2,472/18,513 (13.4%)
Numbers of monitoring examinations per year after 5 years of hydroxychloroquine use, numbers/yr (SD)	0.6 (0.3)	0.6 (0.3)
Mean (SD)/median (IQR) timing of the 1st monitoring examination since hydroxychloroquine use, months	65.9 (3.5)/65.8 (62.9–69.0)	65.6 (3.5)/65.4 (62.5–68.6)
Mean (SD) /median (IQR) interval of monitoring between the 1st and 2nd examinations, months	16.3 (10.3)/12.8 (11.0–18.9)	14.9 (9.6)/12.4 (9.4–17.4)
Mean (SD) /median (IQR) interval of monitoring between the 2nd and 3rd examinations, months	15.1 (7.1)/12.6 (12.1–16.6)	14.4 (6.6)/12.4 (11.7–15.7)
Mean (SD) /median (IQR) interval of monitoring between the 3rd and 4th examinations, months	13.6 (5.1)/12.4 (11.7–14.5)	13.6 (5.1)/12.4 (11.9–14.9)
Modalities used for monitoring (%)		
Optical coherence tomography	3,811 (76.9%)	6568 (81.1%)

© 2023 Kim J et al. *JAMA Network Open.*

Automated visual fields	2,778 (56.0%)	4231 (52.2%)
Fundus autofluorescence	1,719 (34.7%)	2441 (30.1%)
Multifocal electroretinogram	80 (1.6%)	109 (1.3%)

*Patients with DM consists of 37.8% of long-term users during the follow-up period.

© 2023 Kim J et al. *JAMA Network Open.*
