# Supplementary material for: Practice Patterns of Screening for Hydroxychloroquine Retinopathy in South Korea
Source: JAMA Netw Open. 2023 May 23;6(5):e2314816. doi: 10.1001/jamanetworkopen.2023.14816 (PMC10208143; doi:10.1001/jamanetworkopen.2023.14816)
Supplement: Supplement 2. — Data Sharing Statement [file jamanetwopen-e2314816-s002.pdf]

Data Sharing Statement

Kim. Practice Patterns of Screening for Hydroxychloroquine Retinopathy in South Korea. *JAMA Netw Open*. Published May 23, 2023. doi:10.1001/jamanetworkopen.2023.14816

Data
Data available: No

Additional Information
Explanation for why data not available: Data and materials can be requested by e-mail and will be provided after consultation with the IRB.
